# Carriers of rare damaging *CCR2* genetic variants are at lower risk of atherosclerotic disease

**DOI:** 10.1101/2023.08.14.23294063

**Published:** 2023-08-16

**Authors:** Marios K. Georgakis, Rainer Malik, Natalie R. Hasbani, Gabrielle Shakt, Alanna C. Morrison, Noah L. Tsao, Renae Judy, Braxton D. Mitchell, Huichun Xu, May E. Montasser, Ron Do, Eimear E. Kenny, Ruth J.F. Loos, James G. Terry, John Jeffrey Carr, Joshua C. Bis, Bruce M. Psaty, W. T. Longstreth, Kendra A Young, Sharon M Lutz, Michael H Cho, Jai Broome, Alyna T. Khan, Fei Fei Wang, Nancy Heard-Costa, Sudha Seshadri, Ramachandran S. Vasan, Nicholette D. Palmer, Barry I. Freedman, Donald W. Bowden, Lisa R. Yanek, Brian G. Kral, Lewis C. Becker, Patricia A. Peyser, Lawrence F. Bielak, Farah Ammous, April P. Carson, Michael E. Hall, Laura M. Raffield, Stephen S. Rich, Wendy S. Post, Russel P. Tracy, Kent D. Taylor, Xiuqing Guo, Michael C. Mahaney, Joanne E. Curran, John Blangero, Shoa L. Clarke, Jeffrey W. Haessler, Yao Hu, Themistocles L. Assimes, Charles Kooperberg, Scott M. Damrauer, Jerome I. Rotter, Paul S. de Vries, Martin Dichgans

**Affiliations:** 1 Institute for Stroke and Dementia Research (ISD), University Hospital, Ludwig-Maximilians-University (LMU), Munich, Germany; 2 Program in Medical and Population Genetics, Broad Institute of MIT and Harvard, Cambridge, MA, USA; 3 Munich Cluster for Systems Neurology (SyNergy), Munich, Germany.; 4 Human Genetics Center, Department of Epidemiology, Human Genetics, and Environmental Sciences, School of Public Health, The University of Texas Health Science Center at Houston, Houston, TX, USA; 5 Department of Surgery, Perelman School of Medicine at the University of Pennsylvania, Philadelphia, PA, USA; 6 Corporal Michael Crescenz VA Medical Center, Philadelphia, PA, USA; 7 Department of Medicine, University of Maryland School of Medicine, Baltimore, MD; 8 Geriatrics Research and Education Clinical Center, Baltimore Veterans Administration Medical Center, Baltimore, MD; 9 The Charles Bronfman Institute for Personalized Medicine, Icahn School of Medicine at Mount Sinai, New York, NY, USA; 10 Department of Genetics and Genomic Sciences, Icahn School of Medicine at Mount Sinai, New York, NY, USA; 11 The Center for Genomic Health, Icahn School of Medicine at Mount Sinai, New York, NY, USA; 12 Pamela Sklar Division of Psychiatric Genomics, Icahn School of Medicine at Mount Sinai, New York, NY, USA; 13 Department of Medicine, Icahn School of Medicine at Mount Sinai, New York, NY, USA; 14 Department of Radiology, Vanderbilt University Medical Center, Nashville, TN, USA; 15 Cardiovascular Health Research Unit, Department of Medicine, University of Washington, Seattle, WA, USA; 16 Department of Health Systems and Population Health, University of Washington, Seattle, WA, USA; 17 Department of Epidemiology, University of Washington, Seattle, WA, USA; 18 Department of Neurology, University of Washington, Seattle, WA, USA; 19 Department of Epidemiology, University of Colorado Anschutz Medical Campus, Aurora CO, USA; 20 Department of Population Medicine, PRecisiOn Medicine Translational Research (PROMoTeR) Center, Harvard Pilgrim Health Care and Harvard Medical School, Boston, MA, USA; 21 Department of Biostatistics, T.H. Chan School of Public Health, Harvard University, Boston, MA, USA; 22 Channing Division of Network Medicine, Brigham and Women’s Hospital, Harvard Medical School, Boston, MA, USA; 23 Department of Biostatistics, University of Washington, Seattle, WA, USA; 24 Department of Medicine, Boston University School of Medicine, Boston, MA, USA; 25 Boston University and National Heart, Lung, and Blood Institute’s Framingham Heart Study, Framingham, MA, USA; 26 Bigg’s Institute for Alzheimer’s Disease and neurodegenerative disorders, University of Texas Health Science Center, San Antonio, TX, USA; 27 Department of Epidemiology, Boston University School of Public Health, Boston, MA, USA; 28 Department of Biochemistry, Wake Forest School of Medicine, Winston-Salem, NC, USA; 29 Section on Nephrology, Department of Internal Medicine, Wake Forest School of Medicine, Winston-Salem, NC, USA; 30 Department of Medicine, Johns Hopkins University School of Medicine, Baltimore, MD, USA; 31 Department of Epidemiology, School of Public Health, University of Michigan, Ann Arbor, MI, USA; 32 Department of Medicine, University of Mississippi Medical Center, Jackson, MS; 33 Department of Genetics, University of North Carolina at Chapel Hill, Chapel Hill, NC; 34 Center for Public Health Genomics, University of Virginia, Charlottesville, VA USA; 35 Johns Hopkins Bloomberg School of Public Health, Johns Hopkins School of Medicine, Baltimore, MD USA; 36 Departments of Pathology & Laboratory Medicine, and Biochemistry, Larner College of Medicine, University of Vermont, Burlington, VT USA; 37 The Institute for Translational Genomics and Population Sciences, Department of Pediatrics, The Lundquist Institute for Biomedical Innovation at Harbor-UCLA Medical Center, Torrance, CA USA; 38 Department of Human Genetics and South Texas Diabetes and Obesity Institute, University of Texas Rio Grande Valley School of Medicine, Brownsville TX USA; 39 Department of Medicine (Division of Cardiovascular Medicine), Stanford University School of Medicine, Stanford, CA, USA; 40 Stanford Cardiovascular Institute, Stanford, CA, USA; 41 VA Palo Alto Health Care System, Palo Alto, CA, USA; 42 Division of Public Health Sciences, Fred Hutchinson Cancer Center, Seattle WA 98109 USA; 43 Department of Genetics, Perelman School of Medicine at the University of Pennsylvania, Philadelphia, PA, USA; 44 German Centre for Neurodegenerative Diseases (DZNE), Munich, Germany; 46 German Centre for Cardiovascular Research (DZHK, Munich), Munich, Germany

## Abstract

**Background:**

The CCL2/CCR2 axis governs monocyte trafficking and recruitment to atherosclerotic lesions. Human genetic analyses and population-based studies support an association between circulating CCL2 levels and atherosclerosis. Still, it remains unknown whether pharmacological targeting of CCR2, the main CCL2 receptor, would provide protection against human atherosclerotic disease.

**Methods:**

In whole-exome sequencing data from 454,775 UK Biobank participants (40–69 years), we identified predicted loss-of-function (LoF) or damaging missense (REVEL score >0.5) variants within the *CCR2* gene. We prioritized variants associated with lower monocyte count (p<0.05) and tested associations with vascular risk factors and risk of atherosclerotic disease over a mean follow-up of 14 years. The results were replicated in a pooled cohort of three independent datasets (TOPMed, deCODE and Penn Medicine BioBank; total n=441,445) and the effect of the most frequent damaging variant was experimentally validated.

**Results:**

A total of 45 predicted LoF or damaging missense variants were identified in the *CCR2* gene, 4 of which were also significantly associated with lower monocyte count, but not with other white blood cell counts. Heterozygous carriers of these variants were at a lower risk of a combined atherosclerosis outcome, showed a lower burden of atherosclerosis across four vascular beds, and were at a lower lifetime risk of coronary artery disease and myocardial infarction. There was no evidence of association with vascular risk factors including LDL-cholesterol, blood pressure, glycemic status, or C-reactive protein. Using a cAMP assay, we found that cells transfected with the most frequent *CCR2* damaging variant (3:46358273:T:A, M249K, 547 carriers, frequency: 0.14%) show a decrease in signaling in response to CCL2. The associations of the M249K variant with myocardial infarction were consistent across cohorts (OR_UKB_: 0.62 95%CI: 0.39–0.96; OR_external_: 0.64 95%CI: 0.34–1.19; OR_pooled_: 0.64 95%CI: 0.450.90). In a phenome-wide association study, we found no evidence for higher risk of common infections or mortality among carriers of damaging *CCR2* variants.

**Conclusions:**

Heterozygous carriers of damaging *CCR2* variants have a lower burden of atherosclerosis and lower lifetime risk of myocardial infarction. In conjunction with previous evidence from experimental and epidemiological studies, our findings highlight the translational potential of CCR2-targeting as an atheroprotective approach.

## INTRODUCTION

Atherosclerotic cardiovascular disease (CVD) is the leading cause of morbidity and mortality worldwide.^1–3^ Over 20 years of preclinical research have provided overwhelming evidence for a causal role of inflammation in atherogenesis^4,5^ and recent trials provided proof-of-concept that targeting inflammation can lead to reductions in adverse cardiovascular events.^6–8^ The canakinumab anti-inflammatory thrombosis outcome study (CANTOS) demonstrated that a monoclonal antibody against IL-1β lowers risk of recurrent vascular events among individuals with recent myocardial infarction.^6^ The colchicine cardiovascular outcomes trial (COLCOT)^7^ and the low-dose colchicine-2 (LoDoCo2) trial^8^ further showed that colchicine, an established drug with widespread inhibitory effects on inflammatory pathways,^9,10^ lowers the risk of recurrent vascular events in patients with coronary artery disease (CAD).

Targeting inflammation for atheroprotection must be balanced against the impact on any host responses. For example, both canakinumab^6^ and colchicine^7^ were associated with adverse effects including fatal infections in the CANTOS and the colchicine trials. While translational efforts have mostly focused on the inflammasome-IL-1β/IL-6 axis,^11^ ample evidence from preclinical studies and early-phase clinical trials highlights the promise of alternative cytokines^5^ for the development of a second generation of atherosclerosis-centered anti-inflammatory treatments.^4^ CC-motif chemokine ligand 2 (CCL2) is a pivotal inflammatory chemokine regulating monocyte trafficking^12^ that has been intensively studied as a potential target in atherosclerosis. While extensive preclinical data support a causal involvement of CCL2 and its receptor CCR2 in experimental atherosclerosis,^13^ it was not until recently that large-scale genetic and epidemiological studies have highlighted the relevance of the CCL2/CCR2 pathway in human CVD, calling for clinical translation of strategies targeting this pathway.^14^ Indeed, both prospective observational studies^15,16^ and Mendelian randomization analyses from population genetic studies^17,18^ support that higher circulating CCL2 levels are associated with a higher risk of ischemic stroke, coronary artery disease, and cardiovascular death. Furthermore, CCL2 levels are higher in human atherosclerotic lesions derived from patients with symptomatic carotid stenosis, as compared to asymptomatic disease and are associated with features of plaque vulnerability.^19^

While these studies support a key role of the CCL2/CCR2 axis in human atherosclerosis, it remains elusive whether pharmacologically targeting this pathway could lead to atheroprotection in humans. Several molecules targeting CCR2 are currently under development for autoimmune disease, liver disease, and cancer and could be repurposed for prevention of atherosclerotic cardiovascular disease.^14^ Studies examining the phenotypic effects of rare genetic variants in population-based studies have been instrumental in predicting the consequences of pharmacological interventions^20–24^ and might thus serve as a validation step for drug targets under development.

Here, we leveraged whole-exome sequencing data from 454,775 participants of the population-based UK Biobank study to explore whether rare damaging and loss-of-function (LoF) variants in the *CCR2* gene are associated with a lower burden of atherosclerosis and lower lifetime risk of clinical manifestations of atherosclerotic disease. Furthermore, we explored the effects of these variants on monocyte count, as a functional readout and tested associations with traditional vascular risk biomarkers. We aimed to validate the top variant in experimental assays, replicate the findings in external population- and hospital-based biobanks, and explore associations of damaging *CCR2* variants with potential safety signals.

## RESULTS

### *CCR2* variants and associations with monocyte counts

**Figure 1.**
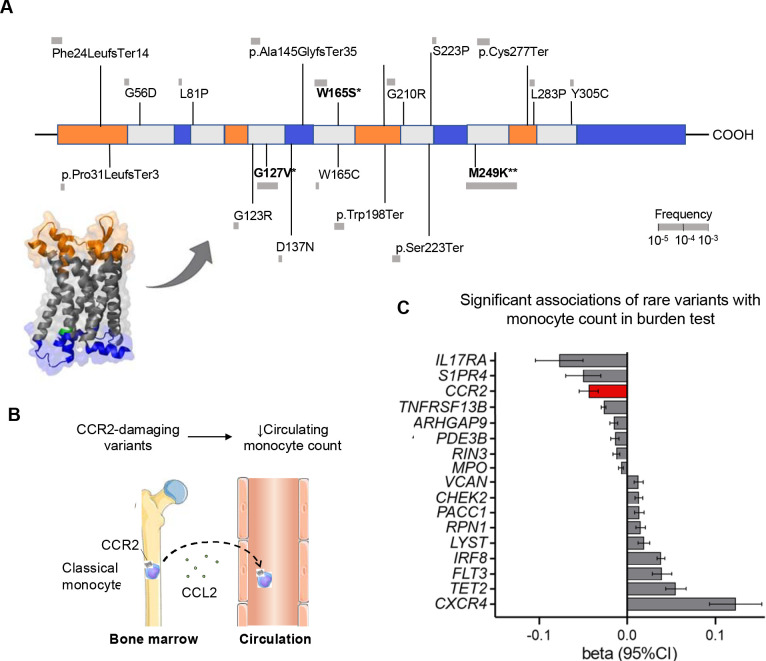
Damaging *CCR2* variants and associations with monocyte count. (**A**) Domain structure of the CCR2 protein and position of the predicted loss-of-function (LoF) or missense damaging (REVEL>0.5) variants present in >2 UK Biobank participants in the *CCR2* exonic regions. (**B**) Schematic of the theoretical predicted effect of the CCL2-CCR2 axis on monocyte recruitment from the bone marrow to circulation. (**C**) Exome-wide gene burden test for monocyte count based on predicted LoF or missense damaging (REVEL>0.5) variants in exonic gene regions. Damaging variants in *CCR2* are associated with lower monocyte count at an exome-wide level.

Among 428,191 unrelated (out of 454,775 with whole-exome sequencing data) UK Biobank participants from the whole-exome sequencing data release, we found a total of 45 predicted LoF or damaging (REVEL>0.5) variants in the exome of the *CCR2* gene distributed across 787 heterozygous carriers (frequency 0.18%, Figure 1A and Supplementary Table S1). There was no homozygous carrier of any of these variants and variants were predominantly prevalent in individuals of European ancestry (779 carriers, frequency 0.20%), as compared to individuals of African, Hispanic or Latin American, East Asian, and South Asian ancestry (total of 8 carriers, pooled frequency 0.02%). Due to the very low frequency in other ancestries, we restricted our analyses to individuals of European ancestry (n=393,416), Baseline characteristics of the study population are presented in Supplementary Table S2.

As expected from the established role of CCR2 in regulating monocyte trafficking from bone marrow to circulation (Figure 1B), *CCR2* was among the top genes showing an association between rare genetic variants predicted to result in a loss of function (LoF) or be damaging and a lower monocyte count in an exome-wide burden test, (Figure 1C and Supplementary Table S3). This validated our approach of using monocyte count as a functional readout for the damaging effect of the *CCR2* variants. Across the 45 tested variants, 4 were also individually associated with lower monocyte count at a p<0.05 (657 carriers, 0.17% pooled frequency), as detailed in Supplementary Table S1.

### Associations of damaging *CCR2* variants with atherosclerosis and vascular risk factors

**Figure 2.**
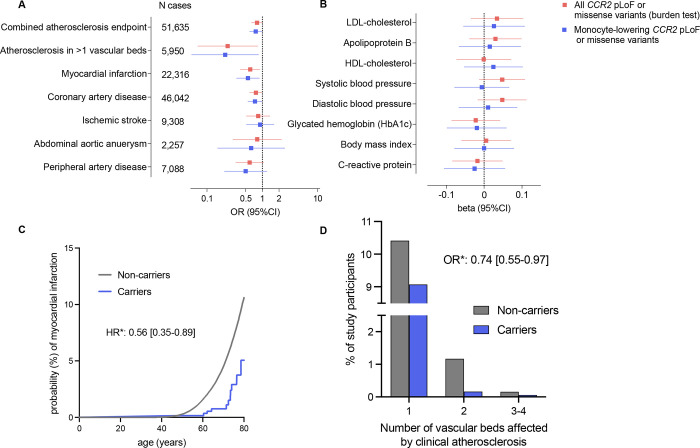
Associations of rare damaging *CCR2* variants with risk of atherosclerotic disease in the UK Biobank. (**A**) Associations of 45 predicted loss-of-function (LoF) or missense damaging (REVEL>0.5) *CCR2* variants (minimum allele frequency <0.01) in a burden test and 4 monocyte-lowering variants within *CCR2* in logistic regression adjusted for age, sex, and the first five principal components with risk of common manifestation of human atherosclerotic disease among UK Biobank participants of European ancestry. (**B**) Associations of 45 predicted LoF or missense damaging (REVEL>0.5) *CCR2* variants in a burden test and 4 monocyte-lowering variants within *CCR2* in linear regression adjusted for age, sex, and the first five principal components with conventional vascular risk factors among UK Biobank participants of European ancestry. HbA1c and C-reactive protein levels were log-transformed for normalization. (**C**) Kaplan-Meier curves for lifetime risk of myocardial infarction among carriers and non-carriers of the 4 monocyte-lowering predicted LoF or missense *CCR2* variants. The hazard ratio (HR) is derived from a Cox proportional hazard model among UK Biobank participants of European ancestry with age as the time variable adjusted for age, sex, and the first 5 ancestral principal components. (**D**) Prevalence of clinically manifest atherosclerosis across 4 vascular beds (coronary arteries, cerebrovascular system, peripheral arteries of extremities, aorta) among carriers and non-carriers of the 4 monocyte-lowering predicted LoF or missense *CCR2* variants. The odds ratio (OR) is derived from an ordinal regression analysis among UK Biobank participants of European ancestry adjusted for age, sex, and the first 5 ancestral principal components.

We next explored whether genetic variation in *CCR2* is associated with atherosclerotic disease. In a two-step approach we first applied a burden test using all 45 predicted LoF or missense damaging variants (REVEL>0.5) in *CCR2* and then prioritized the 4 variants that were also associated with lower monocyte count (Figure 2). With both approaches, we found these variants to be associated with lower risk of a combined atherosclerotic endpoint (burden test: OR: 0.80, 95%CI: 0.65–0.98, p=0.03; monocyte-lowering *CCR2* variants: OR: 0.76, 95%CI: 0.59–0.97, p=0.02), as well as with severe atherosclerotic disease, defined by clinical manifestations in at least two vascular beds (burden test: OR: 0.24, 95%CI: 0.07–0.82 p=0.003; monocyte-lowering *CCR2* variants: OR: 0.21, 95%CI: 0.05–0.85, p=0.009, Figure 2A). We found a trend for a dose-response pattern with lower burden of clinically manifest atherosclerotic disease across the number of vascular beds involved among carriers for the 4 monocyte-lowering *CCR2* variants (frequency of atherosclerosis presence among carriers vs. non-carriers: in no vascular beds 88.1% vs. 90.6%; in 1 vascular bed 10.4% vs. 9.1%; in 2 vascular beds 1.2% vs. 0.2%; in 3 vascular beds 0.3% vs 0.1%, in 4 vascular beds 0.03% vs 0%; OR from ordinal regression: 0.74, 95%CI: 0.55–0.97, p=0.01, Figure 2C).

Across individual endpoints, there were significant associations for myocardial infarction (burden test: OR: 0.60, 95%CI: 0.40–0.90, p=0.008; monocyte-lowering *CCR2* variants: OR: 0.55, 95%CI: 0.34–0.89, p=0.009 for the 4 monocyte-lowering *CCR2* variants) and coronary artery disease (burden test: OR: 0.76, 95%CI: 0.59–0.99, p=0.03, monocyte-lowering *CCR2* variants: OR: 0.71, 95%CI: 0.53–0.96, p=0.01), as well as directionally consistent associations with the odds of all other examined outcomes (ischemic stroke, peripheral artery disease, abdominal aortic aneurysm, Figure 2A). In a Cox regression model using age at occurrence as the time variable, heterozygous carriers of the 4 monocyte-lowering *CCR2* variants were at a 44% lower lifetime risk for myocardial infarction (HR: 0.56, 95%CI: 0.35–0.89, p=0.008, Figure 2D).

To explore whether the effect of the *CCR2* damaging variants is mediated through effects on risk factors targeted by current preventive approaches, we next tested associations with established biomarkers of vascular risk. We found no association between either the full set of the 45 predicted LoF or damaging *CCR2* variants in a burden or the 4 monocyte-lowering variants with any of the tested vascular risk factors including blood pressure, hyperglycemia, or circulating lipids. Furthermore, there was no evidence of association with the levels of the known inflammatory biomarker C-reactive protein, thus suggesting that the effects might be independent of factors targeted by current atheroprotective anti-inflammatory treatments (Figure 2B).

### Functional consequences of *CCR2* damaging variants

**Figure 3.**
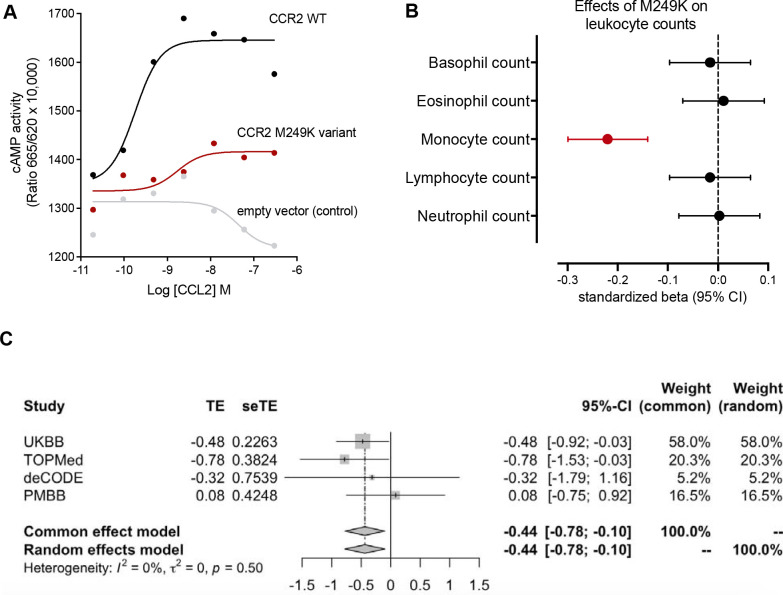
Impact of the most frequent damaging monocyte-lowering *CCR2* variant (3:46358273:T:A, M249K) on CCL2 chemotaxis, monocyte count, and lifetime risk of myocardial infarction. (**A**) Results of cyclic AMP (cAMP) assay. Shown is the cAMP activity in HEK293T cells transfected with either empty vector or wild-type or mutant *CCR2* in response to different concentrations of CCL2. Results are presented as “Ratio 665/620 × 10,000” (ratio of fluorescence at 665 nm and 620 nm × 10,000). (**B**) Associations of M249K (3:46358273:T:A) with counts of different leukocyte populations in the subset of European ancestry participants of the UK Biobank (N=393,838), as derived from linear regression models adjusted for age, sex, and the first 5 ancestral principal components. (**C**) Meta-analysis of the effects of the M249K mutation on risk of myocardial infarction across 4 population- and hospital-based biobanks (851,159 individuals, 657 heterozygous M249K carriers, 67,210 cases). The effects correspond to log-odds ratios derived from logistic regression models adjusted for age, sex, and the first 5 ancestral principal components in each biobank.

As these effects were primarily driven by one variant with the highest frequency (3:46358273:T:A, 89% of all carriers), we prioritized this variant in subsequent functional validation and external replication efforts. This variant has a frequency of 0.15% among UKB participants of European ancestry and leads to the replacement of methionine by lysine in the sixth transmembrane domain of the CCR2 receptor (M249K, Figure 1A). In a cAMP assay, we found that HEK293T cells transfected with CCR2 carrying the M249K variant showed a profound reduction of cAMP production in response to CCL2, when compared to HEK293T cells transfected with the wild-type CCR2 (Figure 3A). Confirming the specificity of the consequences of this variant on monocyte recruitment, we found no evidence of associations with any of the other leukocyte type counts in the UKB (Figure 3B).

### External replication of the effects of M249K

To externally replicate the effects of the M249K *CCR2* variant on risk of myocardial infarction, we meta-analyzed data from three external datasets (TOPMed, deCODE, PMBB). The frequency of the variant varied between the three datasets and was lower than in the UKB (0.05% in the European subgroup of TOPMed, 0.01% in deCODE, 0.10% in the European subset of PMBB), totaling 135 heterozygous carriers among 441,445 individuals (37,526 myocardial infarction cases). When meta-analyzing data from the three cohorts for the association between carrying the variant and risk of myocardial infarction, the OR was comparable to that in the UKB (random-effects meta-analysis: OR: 0.64 95%CI: 0.34–1.19 vs. OR_UKB_: 0.62 95%CI: 0.39–0.96; Supplementary Figure S1 and Supplementary Table S4). When meta-analyzing these data with UKB reaching a sample size of 834,861 individuals including 682 heterozygous M249K carriers (57,500 cases of myocardial infarction), we found carriers of the damaging *CCR2* variant to have 36% lower odds of suffering a myocardial infarction (OR_pooled_: 0.64 95%CI: 0.45–0.90, p=0.009, Figure 3C and Supplementary Table S4).

### Associations with all-cause mortality and risk of infections

**Figure 4.**
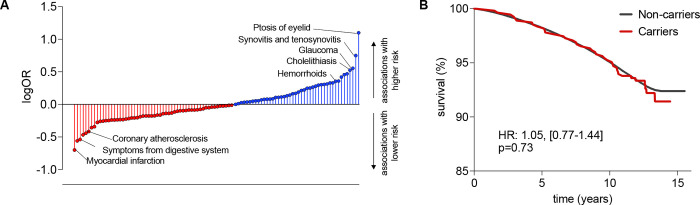
Phenome-wide association study of rare damaging CCR2 variants and associations with overall survival in the UK Biobank. (**A**) Results from phenome-wide association study for 45 predicted loss-of-function (LoF) or missense damaging (REVEL>0.5) *CCR2* variants (minimum allele frequency <0.01) in a burden test among UK Biobank participants of European ancestry. Due to low statistical power, only phecodes with ≥10 cases were analyzed. We present the names of phenotypes associated with rare CCR2 variants at p<0.05. The results are presented as log-Odds Ratios (log-OR). (**B**) Kaplan-Meier curves for overall survival across 15 years of follow-up among carriers and non-carriers of the 4 monocyte-lowering predicted LoF or missense *CCR2* variants in the European subset of UK Biobank participants.

As a last step, we performed a PheWAS in the UKB to explore whether damaging variants in *CCR2* are associated with potential adverse effects that would raise signals for possible side-effects of any CCR2-targeting treatments. Because we lacked statistical power for most of the outcomes, we restricted our analyses to endpoints with ≥10 cases in the carriers group. In an approach pooling all 45 predicted LoF or missense genetic variants in *CCR2*, two of the three phenotypes being less frequent (p<0.05) among carriers were myocardial infarction and coronary atherosclerosis, with myocardial infarction showing the strongest signal (Figure 4A). No infectious disease phenotypes were found to be more common (p<0.05) among *CCR2* damaging variant carriers (Supplementary Table S5). Ptosis of eyelid, tenosynovitis, glaucoma, cholelithiasis, and hemorrhoid disease were more common among carriers. Furthermore, we found no evidence for an association between carrier status for a *CCR2* damaging variants and survival over a 15-year follow-up period among UKB participants (Figure 4B).

## DISCUSSION

Using data from >830,000 individuals we found that heterozygous carriers of rare damaging *CCR2* variants have a significantly lower burden of atherosclerotic disease and a lower lifetime risk of myocardial infarction. Carriers of these variants showed no differences in LDL cholesterol, blood pressure, BMI, HbA1c and C-reactive protein levels, thus supporting that damaging *CCR2* variants exert their effects independently of conventional vascular risk factors targeted by available atheroprotective treatments. Furthermore, we found no evidence of associations with higher risk of infectious diseases or overall mortality among carriers, which could raise safety concerns for a pharmacological approach targeting CCR2. Collectively, our results provide genetic support that targeting the CCL2/CCR2 axis could be a viable strategy for preventing atherosclerotic cardiovascular disease.

Our findings confirm and extend our previous results from a Mendelian randomization study suggesting a higher atherosclerosis risk among individuals with genetic predisposition to elevated CCL2 levels.^17^ That study focused on common genetic variants distributed across the genome and shown to influence CCL2 levels in *trans*. In contrast, the current study assessed damaging rare genetic variants within the *CCR2* gene. The functional impact of M249K, the most frequent variant among those associated with atherosclerotic disease, was experimentally confirmed. As such, our approach is less prone to confounding due to pleiotropic effects of the variants. Despite the lack of data from clinical trials targeting CCR2 in patients with cardiovascular disease, studies in experimental models of atherosclerosis have provided robust evidence for an atheroprotective effect of pharmacologically targeting CCR2.^13^ Our work connects these findings to humans and provides a strong arguments for moving towards a clinical trial with pharmacological targeting of CCR2 in patients with established atherosclerosis.

Interestingly, we found no evidence supporting a connection of *CCR2* damaging variants with established pharmacological targets of atherosclerosis, such as LDL cholesterol, hyperglycemia, or elevated blood pressure. This implies that any benefit from CCR2-targeting approaches would be expected to be independent of available preventive approaches against atherosclerotic disease. Also, we found no evidence of associations with circulating C-reactive protein levels, a biomarker of the activity of the IL-6 signaling pathway that is also the target of atheroprotective anti-inflammatory treatments currently under development.^25^ Based on this, our findings suggest that CCR2 inhibition might have an atheroprotective effect on top of such approaches.

Although our PheWAS analysis was limited by low statistical power, it is noteworthy that we found no evidence for an association of damaging *CCR2* variants with risk of infections, which is a potential barrier to the use of atheroprotective anti-inflammatory treatments. This is in line with previous early phase clinical trials testing inhibitors of CCR2 for other indications, which also found no important safety concerns.^26–28^ Furthermore, we found no evidence of associations with overall survival, minimizing concerns about a significant impact on unknown fatal adverse effects.

Our study has limitations. First, despite leveraging the largest available whole-exome sequencing study, our results for the PheWAS analyses are still limited by low statistical power and should be interpreted cautiously . Given the rarity of the damaging CCR2 variants, any association with rare potential side-effects would ultimately be undetectable in the context of this study. Second, UK Biobank consists primarily of European individuals, and consequently, we detected damaging *CCR2* variants that are predominantly detected in European populations. As such, our results should not be extended to non-European individuals. Third, all analyses are based on individuals heterozygous for the damaging *CCR2* variants, as we found no homozygotes for damaging *CCR2* variants. While this lack of homozygotes can be fully explained by the frequency of damaging *CCR2* variants in the general population, it remains unknown whether homozygous status for damaging CCR2 variants would lead to potentially fatal complications.

In conclusion, we found that heterozygous carriers of damaging *CCR2* variants have a lower burden of clinically manifest atherosclerosis and a lifetime risk of myocardial infarction, that is about 40% lower than in non-carriers. This provides further genetic support for the concept that pharmacological targeting of the CCL2/CCR2 axis could be an efficient and viable strategy to prevent atherosclerotic cardiovascular disease.

## ONLINE METHODS

### Study population

We used data from the UK Biobank (UKB) study, a population-based prospective cohort study of UK residents aged 40–69 years recruited between 2006–2010 from 22 assessment centers across the UK.^29^ This analysis was restricted to 454,775 out of 502,419 participants with available whole-exome sequencing data (UK Biobank Exome 450k release from October 2021). Primary and secondary analyses were performed with an updated Functional Equivalence (FE) protocol that retains original quality scores in the CRAM files (referred to as the OQFE protocol). We included only variants that met published criteria^30^: individual and variant missingness <10%, Hardy Weinberg Equilibrium p-value>10^−15^, minimum read coverage depth of 7 for SNPs and 10 for indels, at least one sample per site passed the allele balance threshold >0.15 for SNPs and 0.20 for indels. We used genotype array data released by the UKB study to assign individuals to continental ancestry super-groups (African (AFR), Hispanic or Latin American (HLA, originally referred to as ‘AMR’ by the 1000 Genomes Project), East Asian (EAS), European (EUR) and South Asian (SAS)) by projecting each sample onto reference principal components (PCs) calculated from the 1000 Genomes reference panel. In brief, we merged our samples with all 1000 Genomes samples and kept only SNPs with MAF>10%, genotype missingness<5%, and Hardy–Weinberg equilibrium test P < 10^−5^ that were common between the two datasets. Following pruning to approximately 200,000 independent variants, we calculated PCs for the 1000 Genomes samples using flashpca2^31^ and projected each of the UKB samples onto those PCs. To assign a continental ancestry group to each non-1000 Genomes sample, we trained a random forest model using the 1000 Genomes PCs and calculated the likelihood of a given sample belonging to each of the five continental ancestry groups. When the likelihood for a given ancestry group was >0.9, the sample was assigned to that ancestry group. Otherwise, the sample was put into the group “other/admixed”. We included only individuals without evidence of relatedness within the UKB samples, as defined by a KING cut-off of <0.084.

### Detection of damaging genetic variants in *CCR2* in UK Biobank

One of the key functions of the CCL2 axis is the recruitment of classical monocytes from the bone marrow to the circulation in a CCR2-dependent way.^32–34^ Consequently, damaging variants in the *CCR2* gene would be expected to be associated with a lower monocyte count in the circulation. Restricting our analyses to the *CCR2* gene, we selected damaging rare variants in a two-step process. First, we detected predicted loss-of-function (LoF) or damaging missense variants with a MAF<1% in the *CCR2* exomic region and then explored, which of those are associated with monocyte count as a functional readout. Variants from WES were annotated as previously described^35^ using VEPv101.^36,37^ We used the LofTee plugin for predicting LoF variants^38^ and a REVEL cutoff of > 0.5 from dbNSFP version 4.0a for predicted damaging missense mutations.

To test associations with the circulating counts of monocytes and other white blood cells (WBC), absolute counts were extracted from the UKB fields 30160 (basophil counts), 30150 (eosinophil counts), 30130 (monocyte counts), 30120 (lymphocyte counts) and 30140 (neutrophil counts). Distributions were visually checked for normal distribution and log-normalized when needed. Associations were tested using regenie v2.2.4.^39^ For WBC analyses, we used sex, age at blood draw, and the first 5 ancestral PCs as covariates. The mixed model parameters were estimated using 200,000 genotyped common variants. Saddle point approximation regression was applied. Genetic variants associated with monocyte count at a p-value<0.05 were selected for further analysis. To test whether the associations are specific for monocyte count, we also tested associations with other WBC counts (basophils, eosinophils, lymphocytes, neutrophils) for each variant.

### Exome-wide burden test for monocyte count

To confirm our hypothesis that monocyte count would be lower due to LoF or damaging *CCR2* variants, we performed an exome-wide burden test to explore whether rare genetic variation in *CCR2* is associated with lower monocyte count. For each gene, we considered a burden of rare predicted LoF and damaging missense variants with MAF≤1%, as defined above. We used all available burden tests integrated in regenie (SKAT, SKATO, SKATO-ACAT, ACATV, ACATO, ACATO-FULL). We used the GrCh38 refFlat definition of genes as provided by the UCSC genome annotation database. Significant results were selected on the basis of a Bonferroni-corrected p-value threshold of 2.7×10^−6^ (0.05/18,087 tested genes).

### Associations with atherosclerotic outcomes and vascular risk factors

To explore whether damaging variants at *CCR2* are associated with atheroprotection, we tested associations with atherosclerotic phenotypes. We constructed phenotypes of atherosclerosis in 4 vascular beds (coronary arteries, cerebrovascular system, peripheral arteries of the extremities, and aortic atherosclerosis) using ICD-10- and ICD-9- coded diagnoses, OPCS4-coded procedures, self-report, and algorithmically-defined phenotypes provided by the UKB, as detailed in Supplementary Table S6. We combined these clinical manifestations in a combined atherosclerosis phenotype and defined severe atherosclerosis as presence of clinical manifestations in at least two vascular beds. Furthermore, we explored associations with individual phenotypes of interest including myocardial infarction, coronary artery disease, acute ischemic stroke, peripheral artery disease, and abdominal aortic aneurysm (Supplementary Table S6).

We followed two approaches to test associations with these phenotypes: (i) we performed a burden test combining all predicted damaging variants (LoF or REVEL >0.5) within the *CCR2* gene; (ii) we explored associations of carrying any of the 4 monocyte-lowering *CCR2* damaging variants in a logistic regression model adjusted for age, sex, and the first 5 ancestral PCs. We included both prevalent and incident endpoints as outcomes in all analyses and used Firth’s correction for unbalanced case/control ratios in our logistic regression analysis. To explore whether the associations with atherosclerotic endpoints are mediated through known vascular risk factors, we further tested associations of the damaging genetic variants with the following phenotypes: systolic and diastolic blood pressure, circulating LDL and HDL cholesterol, circulating apolipoprotein B levels, circulating glycated hemoglobin A1c (HbA1c) concentration, body mass index, and C-reactive protein. Individuals under antihypertensive medications were excluded in the analyses for 18ystoleic and diastolic blood pressure, individuals under lipid-lowering medications were excluded from the analyses for LDL and HDL cholesterol and apolipoprotein B levels, and individuals under glucose-lowering treatments were excluded from the analyses for HbA1c.

### Validation of M249K *CCR2* variant in external datasets and meta-analysis

For external validation, we requested summary statistics from three different data sources based on a pre-defined protocol: the TransOmics and Precision Medicine Program supported by NHLBI (TOPMed) Program including multiple studies and a trans-ancestry population in the US (n=51,732), the population-based deCODE dataset in Iceland (n=345,992), and the hospital-based Penn Medicine Biobank (PMBB, n=43,721). Specifically, we requested data from logistic regression models adjusted for age, sex, race, study-specific variables (e.g. sequencing center) and the first 10 PCs (with the Firth’s correction) for the 4 monocyte-lowering *CCR2* variants. The derived odds ratios (OR) from the three datasets were meta-analyzed using fixed-effects and random-effects meta-analyses and were subsequently also meta-analyzed with the results from the UKB. Heterogeneity was assessed with the I^2^ and the Cochran Q statistic.

The Trans-Omics for Precision Medicine (TOPMed) program sponsored by the National Heart, Lung and Blood Institute (NHLBI) has generated deep whole genome sequencing (WGS) data in over 80 participating cohorts and over 150,000 participants. TOPMed whole geneome sequencing methods have been described fully previously^40^. Briefly, WGS was performed at the designated sequencing center for each included study. The sequencing data was aligned to human genome build GRCh38, and basic quality control measures were performed by the TOPMed Informatics Research Center. The data were subsequently released in different freezes. This analysis used Freeze 10. A total of 184,878 TOPMed, CCDG and 1000G samples underwent ≈30× WGS using DNA extracted from blood samples at designated sequencing centers for variant discovery. Basic quality control procedures were implemented by the TOPMed Informatics Research Center.

Of the 80 participating cohorts, 12 cohorts (Supplemental Table S7) have data on myocardial infarction and were made available for this study (n= 6,425 cases and 45,307 controls). TOPMed phenotype data was harmonized by the TOPMed Atherosclerosis Working Group to create prevalent coronary artery disease cases and control for each cohort. Cases were defined as individuals who experienced acute myocardial infarction according to hospital records or self-report. Controls were defined as non-cases who did not have documented angina, coronary artery revascularization and did not have possible coronary artery disease related death. One cohort, BioMe, had a high prevalence of peripheral arterial disease (PAD). Individuals with PAD were also removed from being considered a control. TOPMed WGS and phenotype data are available on dbGAP (study access numbers in Supplemental Table 1)).

The analysis was performed on Encore, a web-based tool to conducted large scale association testing with TOPMed sequencing data, using a Saige Logistic Mixed Model accounting for genetic relatedness through a kinship matrix (https://doi.org/10.1038/s41588-018-0184-y). The model adjusted for the following covariates: sex, self-identified race/ethnicity, TOPMed cohort, sequencing center, and ancestral principal components (PCs) 1 through 10. PCs were computed using PLINK (http://pngu.mgh.harvard.edu/purcell/plink/).

#### DeCODE study

Icelandic myocardial infarction cases were identified from a registry of individuals diagnosed at Landspitali University Hospital in Reykjavik, the only tertiary referral center in Iceland, during the years 1981 to 2022. The criteria for Myocardial infarction diagnosis were defined as previously described in Helgadottir et al, 2007^41^ and genotyping and statistical analysis as described in Aegisdottir et al. 2023.^42^

#### Penn Medicine Biobank

The Penn Medicine Biobank (PMBB) is an academic healthcare system-based genomic and precision medicine cohort that links participant blood and tissue samples with associated health information. Procedures for recruitment, consent, data collection and genotyping are detailed elsewhere.^43^ Individuals of European ancestry with available whole-exome sequencing data were included in the analysis (n=43,723). Among whole exome variants in the CCR2 gene, we identified a total of 14 predicted LOF or potentially deleterious (REVEL > 0.5) variants, distributed across 68 heterozygous carriers and no homozygous carriers. Association testing against outcomes of interest was performed using Firth’s penalized logistic regression - adjusting for age, sex, and the first 5 principal components of ancestry. Myocardial infarction was defined as previously described (Pan-UKB team: https://pan.ukbb.broadinstitute.org 2020).

### Experimental validation of M249K as a damaging variant

Human HEK293T cells were lifted with non-enzymatic cell stripper and resuspended in assay buffer at desired concentrations. Cyclic AMP (cAMP) assays were performed according to the manufacturer’s protocol using the MULTISCREENTM TR-FRET cAMP 1.0 No Wash Assay Kit (Multispan, Inc., Cat# MSCM01). The cells were treated with the cognate ligand of CCR2, CCL2, reconstituted to 30 uM concentration in sterile 0.1% BSA PBS, followed by addition of forskolin and incubation at 37 °C for 20 minutes. The reaction was terminated by sequentially adding MULTISCREEN^™^ Eu-labeled cAMP and MULTISCREEN^™^ 650-labeled anti-cAMP antibody in lysis buffer. The plate was then incubated at room temperature for 30 minutes before reading fluorescent emissions at 620 nm and 665 nm with excitation at 314 nm on FlexStation III (Molecular Devices). The HEK293T cells stably expressing CCR2, HEK293T parental cells, and transiently transfected mutant CCR2 pools were used in the assay. Cells were stained with an anti-FLAG antibody at 2 μg/mL at 4°C for 1 hour in the dark, followed by 3 washes in PBS plus 0.1% BSA and 0.2% Na Azide before being analyzed on a flow cytometer for surface receptor expression. 3,000 events were collected for each sample and the data were analyzed using CellQuest Pro (Becton Dickinson). The cAMP assay results are presented as “Ratio 665/620 × 10,000” (ratio of fluorescence at 665 nm and 620 nm × 10,000).

### Phenome-wide association study

To explore potential adverse effects associated with damaging *CCR2* genetic variants, we tested associations with the full range of clinical phenotypes encoded in the UKB. We used the Phecode Map 1.2 to map UKB ICD10-codes to Phecodes^44^ using all ICD10 codes (main position, secondary position, death records) from the UKB. We excluded Phecodes with <100 cases and Phecodes that are male- or female-specific. Individuals were assigned a case status if >1 ICD10 code was mapped to the respective Phecode. To approximate effect size in a logistic regression framework, we used minor allele carrier status as an independent variable and age at baseline, sex and 5 ancestry PCs as covariates. We used Firth’s correction for unbalanced case/control ratios in our logistic regression analysis for all results with p<0.05.

### Ethics and data availability

All studies have received ethical approvals from the respective ethical authorities and participants in all studies have provided written informed consent. Data from the UKB are available upon submission and approval of a research proposal. The UKB has institutional review board approval from the Northwest Multi-Center Research Ethics Committee (Manchester, UK). We accessed the data following approval of an application by the UKB Ethics and Governance Council (Application No. 36993 and 2532). Summary results from deCODE, TOPMed, and PMBB were obtained following a common pre-defined research protocol to principal investigators of the study and are presented in the figures and supplementary tables. deCODE has been approved by the National Bioethics Committee of Iceland, and the study was conducted in agreement with conditions issued by the Data Protection Authority of Iceland (VSN_14-015). All common research protocols for the TOPMed Program have been approved by the institutional review board at the University of Maryland Baltimore, whereas individual participating studies have obtained ethical approval from their local ethical authorities, as described previously.^45^ The Penn Medicine BioBank is approved by the University of Pennsylvania.

## Supplementary Material

Supplement 1

Supplement 2
