# Supplementary material for: Carriers of rare damaging CCR2 genetic variants are at lower risk of atherosclerotic disease
Source: medRxiv. 2023 Aug 16:2023.08.14.23294063. Preprint. [Version 1] doi: 10.1101/2023.08.14.23294063 (PMC10462211; doi:10.1101/2023.08.14.23294063)
Supplement: Supplementary file 1 [file media-1.docx]

**Supplementary Figure S1.** Meta-analysis of three external cohorts for the effect of the M249K variant on risk of myocardial infarction (N=457,743, 47,236 cases, 110 M249K *CCR2* carriers). All cohorts are adjusted for age, sex, and the first 5 ancestral principal components.
